# Phosphorus Knowledge and Dietary Intake of Phosphorus of US Adults Undergoing Dialysis

**DOI:** 10.3390/nu16132034

**Published:** 2024-06-27

**Authors:** Sydney T. Schneider, Alexander Klug, Jeanette M. Andrade

**Affiliations:** 1 UCF College of Medicine, University of Central Florida, Orlando, FL 32827, USA; sy922022@ucf.edu; 2 USF Health Morsani College of Medicine, University of South Florida, Tampa, FL 22602, USA; kluga@usf.edu; 3 Food Science and Human Nutrition Department, University of Florida, Gainesville, FL 32611, USA

**Keywords:** peritoneal dialysis, hemodialysis, dietary phosphorus, phosphorus knowledge

## Abstract

Abnormal serum phosphorus is a concern for adults undergoing dialysis due to the risk for mortality and morbidity. General recommendations for maintaining serum phosphorus within normal limits is monitoring dietary intake of phosphorus and taking phosphate binders, as prescribed. However, limited research is available about adults’ phosphorus knowledge and dietary intake of phosphorus. The purpose of this cross-sectional study was to determine the association between phosphorus knowledge and dietary intake of phosphorus of adults on dialysis. An online Qualtrics survey was conducted during February–September 2023. Participants (*n* = 107) responded to the 74-item questionnaire (30-day food frequency questionnaire, phosphorus knowledge questionnaire, and demographic questions). Analysis included frequencies, descriptive statistics, t-tests, and Spearman correlations. JMP SAS v16 was used with a statistical significance of *p* < 0.05. Of the participants, 57.0% (*n* = 61) were on peritoneal dialysis and 43.0% (*n* = 46) were on hemodialysis. Average phosphorus knowledge score was 10.6 ± 3.0 out of 19 or 55.8%, with those on peritoneal dialysis having lower scores (54.7%) compared to participants on hemodialysis (58.1%) (*p* < 0.05). The daily average dietary phosphorus intake was 605 ± 297 mg. Participants on peritoneal dialysis consumed more phosphorus (625 mg) compared to participants on hemodialysis (576 mg) (*p* < 0.05). There was no association with phosphorus knowledge scores and dietary intake of phosphorus. There were positive correlations between discussing about phosphorus, knowing serum phosphorus concentration, and phosphorus knowledge scores. These results can aid practitioners in providing tailored nutrition education among adults on dialysis.

## 1. Introduction

Chronic kidney disease (CKD) is defined as an abnormality in either kidney function or structure that is present for at least 3 months [1]. It is estimated that 15% or 37 million adults in the US have CKD, with close to one in nine not knowing that they have the disease [2]. CKD is more common in adults aged 65 years and older and in those who identify as African-American and Hispanic [2]. The progression of CKD can be monitored via staging, which involves an assessment of an individual’s estimated glomerular filtration rate (eGFR) and albuminuria [3]. For individuals with end-stage kidney disease (ESKD), characterized by an eGFR of ≤15 mL/min, a kidney transplant or dialysis such as peritoneal dialysis (PD) and hemodialysis (HD) are common treatment options [4,5]. As of 2020, approximately 558,000 of the 808,000 US individuals who were on dialysis, 84.2% received HD (in-center or at home), 12.7% received PD, and 3.1% awaited a kidney transplant [6]. While it is not agreed whether PD or HD leads to better survival outcomes [5], both methods improve longevity and quality of life [4].

For adults undergoing dialysis, monitoring of diet is essential, especially with phosphorus, as high serum phosphorus concentrations can contribute to cardiovascular and cerebrovascular events and ultimately death [7,8]. In humans, serum concentrations of phosphate are controlled by a variety of mechanisms, including bone flux, absorption of dietary phosphorus, and urinary excretion [7,9,10]. In an adult without CKD, the clinical standard range of phosphorus is between 3.0 mg/dL to 4.5 mg/dL; however, this value can be elevated, 5.5 mg/dL or higher, for those with later stages of CKD [8]. This biological phenomenon, often referred to as hyperphosphatemia [9,11], occurs in adults on dialysis, as their glomerular filtration rate (GFR) is too low to filter phosphate out of the blood and they also show a sustained increase in parathyroid hormone that ultimately results in increased levels of phosphate released into the blood from bone tissue [8,9,10,11]. Furthermore, hyperphosphatemia may occur related to difficulty in adhering to the use of phosphate binders (which can distort taste and can be difficult to swallow), and inadequate protein and phosphorus intake [12].

For adults on dialysis, the recommended dietary allowance of phosphorus is 1000 mg per day; however, due to the abundance of phosphorus in many commonly consumed foods [9,10,12], intake is generally more [12]. Foods can contain organic or inorganic phosphorus. Forty-five percent of the diet is comprised of the inorganic form of phosphorus such as cakes/pies, cheese, and soft drinks with 20% of the diet coming from the organic form of phosphorus like chicken, milk, and egg [13]. The organic form of phosphorus can be 60% to 80% absorbed, whereas the inorganic form of phosphorus can be absorbed at a level of 90% or higher [12]. Identifying the foods that adults on dialysis consume is a key step in maintaining dietary phosphorus recommendations, and in effect serum phosphorus concentrations [12]. Generally, education about phosphorus is provided to adults on dialysis that may include dietary recommendations for phosphorus, foods that contain phosphorus, phosphorus binders, and others. However, the relationship between adults’ phosphorus knowledge and dietary intake of phosphorus is not known. The purpose of this cross-sectional study was to determine the associations between phosphorus knowledge scores and other demographic factors such as dialysis vintage, type of dialysis, race, education level, and total household income and dietary intake of phosphorus of adults on dialysis. It was hypothesized that there would be a negative relationship between phosphorus knowledge scores and dietary intake of phosphorus, in which high phosphorus knowledge scores would contribute to meeting at or consuming below dietary phosphorus recommendations.

## 2. Materials and Methods

### 2.1. Study Design

A cross-sectional observational online study was employed from February–September 2023. Participants were recruited from Facebook and Reddit groups such as “Peritoneal Dialysis Support Group”, “Home Peritoneal Dialysis”, “Dialysis and Kidney Disease”, “Dialysis Discussion Uncensored”, Dialysis Group”, and “Kidney Dialysis Group”. A user-friendly approach was used, which often included posting a message in an online thread with a request for those who fit the study criteria to complete the surveys and questionnaires. ResearchMatch [14] was also used to only recruit participants who had self-identified as being on PD or HD. These recruitment criteria were implemented to establish a sufficient sample size while also excluding those for whom the questionnaires would not be relevant. The inclusion criteria included adults who were 18 years of age or older and were on PD or HD as self-reported. Participants who did not meet these criteria were excluded. A priori power analysis [15] was used to estimate the sample size based on the correlation between phosphorus knowledge scores and dietary intake of phosphorus using a medium effect size of 0.03, an alpha of 0.05, and a power of 80%. A total sample size of 64 participants would be adequate for determining this correlation. 

Participants who were deemed eligible and consented, completed the questionnaire, which included the phosphorus knowledge questions along with a 30-day food frequency questionnaire and demographic questions through Qualtrics, an online survey platform. The survey was determined exempt by the University of Florida Institutional Review Board (IRB) on 6 January 2023.

### 2.2. Stage 1: Development of the Phosphorus Knowledge Questionnaire

The phosphorus knowledge questionnaire was developed and validated at a Southeastern University. The development of this questionnaire followed a two-stage process. Stage 1 involved the development of the phosphorus questions and review of the questions for face and content validity with content matter experts. Stage 2 involved a pilot study with individuals on PD to assess validity. 

Initially, the literature was reviewed for similar knowledge questionnaires that were focused on individual nutrients [16,17,18]. The first version of the phosphorus questionnaire included a total of 20 items. Three items inquired if a health professional discussed phosphorus in participants’ diets, frequency of providing phosphorus information, and the types of information provided (e.g., pamphlets). The remaining 17 items focused on phosphorus in foods such as dairy being a rich source of phosphorus and reading food labels (7 items). Other items focused on the impact phosphorus has on the human body and health and the dietary recommendation for phosphorus [19]. Content matter experts—a dialysis nurse (*n* = 1), a social worker (*n* = 1), and renal dietitians (*n* = 2)—reviewed the questionnaire for face and content validity. The experts suggested reducing the number of dietary phosphorus and mechanism questions and including other questions that pertained to participants’ knowledge of their serum phosphorus level and their use of phosphate binders. If participants indicated that they used phosphate binders, supplementary questions were included about the frequency of taking the binders and knowledge of their respective phosphate binder [20]. One question was removed altogether due to non-relevance to the population. The final questionnaire consisted of 19 questions.

### 2.3. Stage 2: Validation

A pilot study was conducted from February until March 2023 with participants who were on PD. This was to test the ease and accessibility of the survey. Participants were included in this study if they were over the age of 18, were able to read and understand English, and were on PD for at least one month. This pilot study initially included a total of 30 participants. No participants indicated challenges with the survey questions, thus recruitment opened for additional PD and HD participants. Initially, a total of 114 participants agreed and consented to the study. No phosphorus education from the researchers took place as the intention was to assess their phosphorus knowledge based on if information was provided to them and the type. For the final analysis, seven were excluded due to incompleteness of the survey—not completing either the phosphorus knowledge questions or the food frequency questionnaire.

### 2.4. Food Frequency Questionnaire

To align the phosphorus knowledge responses to dietary intake of phosphorus, participants completed a modified version of the validated Dana Farber’s Cancer Institute Eating Habits Questionnaire [21]. Participants responded to the frequency of consuming 62 food and beverage items over the past 30 days to reduce potential recall bias. The food items were split into seven subgroups: dairy (7 items), fruits (6 items), vegetables (10 items), seafood (9 items), meat (8 items), sweets, baked goods, cereal and miscellaneous (13 items), and beverages (9 items). The available answer options for each food product included: never or less than once per month, 1–3 times per month, 1 time per week, 2–4 times per week, 5–6 times per week, 1 time per day, 2–3 times per day, 4–5 times per day, and 6 or more times per day. To determine total phosphorus consumed daily, every answer choice ranging from “Never or less than once per month” to “6 or more times per day” was assigned a value that equates to the number of servings consumed in one day. The values used included: 0 (never or less than once per month), 0.07 (1–3 times per month), 0.14 (1 time per week), 0.43 (2–4 times per week), 0.79 (5–6 times per week), 1 (1 time per day), 2.5 (2–3 times per day), 4.5 (4–5 times per day), and 6 (6 or more times per day). Then, the results for each food item were multiplied by the amount of phosphorus in one serving to determine the amount of phosphorus consumed from each food item in a day. The sum of these values was then obtained, equating to the participant’s average daily phosphorus consumption as per the methods used from other studies [22,23].

### 2.5. Demographic Questions

Demographic questions included dialysis vintage, type of dialysis, race, highest education level, and total household income. For questions pertaining to race, education level, and income, participants were provided the answer choice of “prefer not to say”. Questions regarding gender and age were not included in the questionnaire as previous studies observed that neither gender [24,25,26] nor age [24] had significant associations with nutritional knowledge in the dialysis community. Furthermore, as this was a self-administered survey and chart reviews of the participants’ data were not accessible, no collection of co-morbidities, blood or urine, compliance with dialysis, or dialysis prescription took place.

### 2.6. Analysis

For the knowledge scores, for every correct response a score of 1 was provided and incorrect or ‘I do not know’ responses received a score of 0. The scores were then summed for a total of 19. The total scores were divided by 19 and converted to a percentage to obtain their overall phosphorus knowledge percentage score. The knowledge score was interpreted as the number of correct answers, with >75% indicating good knowledge, 50–75% indicating average knowledge, and <50% indicating inadequate knowledge [27,28]. Descriptive statistics were obtained for the study variables. Mean and standard deviations were reported for the continuous variables (phosphorus knowledge and dietary intake of phosphorus), and frequency was reported for the categorical and dichotomous variables (race/ethnicity, income status, dialysis vintage, and highest education level). Normality was assessed visually by histograms/QQ plots. Independent t-tests were used to compare differences in continuous variables for participants who were on PD compared to participants on HD for continuous variables (phosphorus knowledge and dietary intake of phosphorus). A Pearson correlation was conducted to observe associations between the continuous variables—phosphorus score and dietary intake of phosphorus. Pearson and Spearman correlations were conducted to observe associations between the continuous, categorical, and dichotomous variables being studied. JMP SAS v16 [SAS Institute Inc., Cary, NC, USA] was used with a statistical significance of *p* < 0.05.

## 3. Results

### 3.1. Participant Demographics

From the final analysis of 107 participants, 56.1% (*n* = 60) were on PD and 43.9% (*n* = 47) were on HD. Participants were included in the analysis even if they did not respond to all demographic questions; thus, for some questions no responses were collected from a portion of participants. The dialysis vintage of participants was highest at 1–2 years (26.4%) or 4 years or more (25.5%), identified as White (77.9%), and had some college or had graduated from a 4-year bachelor’s (*n* = 58 or 54.2%). About 50% of participants had a household income of 74,999 USD or less. Furthermore, 95.3% of participants had education about phosphorus in their diet, with 56.1% of participants indicating that these phosphorus conversations occurred monthly. Based on chi-square analyses, statistical differences were observed with dialysis vintage (*p* = 0.02) but no other demographic variables were dependent on if a participant was on PD or HD (see Table 1).

### 3.2. Phosphorus Knowledge

The mean total phosphorus knowledge score of all participants was 10.6 ± 3.0 out of 19 or 55.8%. Based on the results of the independent t-test, participants on HD, on average, scored significantly higher (11.0, CI 95% [10.2, 11.9]) compared to participants on PD (10.4, CI 95% [9.7, 11.1], *p* = 0.00) (see Table 2). Of the 84% who indicated they took a phosphate binder, 86% indicated they took a phosphate binder with every meal unless it was fruit, 8.9% said they took a phosphate binder once daily, and 3.3% took a phosphate binder once a week. Twenty-six percent (*n* = 29) of participants did not know their exact serum phosphorus concentrations yet participants were aware if their serum phosphorus was abnormal or not.

### 3.3. Dietary Intake of Phosphorus

Across all participants (*n* = 107), the mean daily dietary intake of phosphorus was 605 ± 297 mg. An independent *t*-test illustrated that participants in the PD group had a higher daily average phosphorus intake (625 mg) compared to participants in the HD group (570 mg) (*p* < 0.00) (see Table 2). The highest percentage of daily phosphorus intake was from the sweets, baked goods, cereals, and miscellaneous category (28.1%), followed closely by the meat category (28.0%). Participants consumed less of their daily phosphorus intake from seafood (7.0%), fruit (4.4%), and the least amount of phosphorus from beverages (4.3%). The food item consumed the most was fresh fruit (including pears, apples, peaches, etc.) with an average of 0.75 servings per day. 

### 3.4. Correlation Results

Based on a Pearson correlation, there was no statistical difference among phosphorus knowledge scores and dietary intake of phosphorus (r = 0.05, *p* = 0.78). Based on a Spearman correlation, there were statistically significant positive correlations among phosphorus knowledge scores with having received education about phosphorus (r = 0.21, *p* < 0.05) and knowledge of serum phosphorus (r = 0.44, *p* < 0.001) (see Table 3). No other significance among variables was observed.

## 4. Discussion

This cross-sectional study examined the associations between phosphorus knowledge, dietary intake of phosphorus, and demographic factors among participants on PD and HD. Overall, phosphorus knowledge was higher in participants on HD compared to those on PD, whereas dietary intake of phosphorus was higher in PD participants compared to HD. There was no association between phosphorus knowledge and dietary intake of phosphorus regardless of covariates, yet there were positive associations between phosphorus knowledge scores and having discussed phosphorus and knowing serum phosphorus concentrations. 

Results of the study demonstrated that participants on PD, on average, scored lower on the phosphorus knowledge questionnaire than participants on HD. This finding may be due to differences in the frequency of medical visits and check-ups between the two treatment options. Adults on HD attend dialysis sessions at least three times weekly [29], thus they may be exposed to medical professionals more often than adults on PD, who often attend clinics monthly [30]. However, questions pertaining to the number of sessions and frequency of attending these sessions were not included on the survey, thus future research should explore this factor. Possibly adults on HD may be able to ask for more input or be exposed to information more often compared to those on PD. Moreover, there was a difference in dialysis vintage whereby participants on HD were on it longer than participants on PD. This may have been attributed to more retention of information due to the length of times being presented with it. This was similar to a cross-sectional study among adults on HD (*n* = 218) that showed that more years on dialysis contributed to a higher health literacy and dietary adherence [31]. A significant positive correlation was observed between phosphorus knowledge scores and having discussed about phosphorus. This likely resulted in these participants acquiring more knowledge about phosphorus in general and being more aware of the foods they are consuming or phosphorus presence in foods. 

Elevated serum phosphorus in adults undergoing dialysis increases the risk for cardiovascular and cerebrovascular events [7,9]. The mean daily dietary intake of phosphorus across all participants was 605 ± 297 mg, which is below the recommendation of 1000 mg for those on dialysis [3,19]. Results of the study demonstrated that participants in the PD group had a higher daily average phosphorus dietary intake compared to participants in the HD group. This difference may be due to the observed lower average phosphorus knowledge score of participants undergoing PD compared to participants undergoing HD, indicating that adults on PD may not be as informed about phosphorus and its impact on kidney health. This lower phosphorus knowledge may lead to lower vigilance and attention paid towards monitoring phosphorus dietary consumption, ultimately leading to higher average phosphorus consumption compared to those undergoing HD. This is in opposition to another study that illustrated that adults on PD (*n* = 30) consumed less phosphorus compared to adults on HD (*n* = 30). This was mainly attributed to consuming less than three meals daily [32]. Even though this current study did not include the number of meals participants consumed, possibly participants were consuming less meals than recommended that could have attributed to this difference. 

Among all participants, the highest percentage of daily phosphorus intake was from sweets, baked goods, cereals, miscellaneous items, and meat items. At least for sweets, baked goods, cereals, and miscellaneous items (e.g., condiments), they are often pre-packaged and processed, with inorganic phosphates added to increase their shelf-life [19]. Inorganic phosphates have a higher bioavailability because they are not bound to proteins [19]; thus, increased consumption of inorganic phosphates can lead to higher serum phosphorus concentrations compared to consumption of organic phosphates [8,9]. In general, those adhering to a diet with the goal of preserving kidney functions must balance not only phosphorus intake, but also the consumption of other minerals and nutrients. It is generally recommended for adults on dialysis to monitor the consumption of potassium, sodium, calcium, and protein in foods [12,33]. Many of these recommendations can restrict foods that are high in organic phosphates, including foods such as animal-based and plant-based proteins. This restriction may inadvertently cause an increased reliance on foods with high levels of inorganic phosphate, resulting in the participants’ high contribution to the total daily phosphorus intake in the study. Additionally, diminished appetite is a common symptom for those undergoing both HD and PD [34,35,36], and may result in those on dialysis consuming more calorically dense, processed foods to meet energy requirements while limiting the volume of food consumed.

Over a quarter of participants did not know their serum phosphorus concentrations; however, there was no observable difference in the types and frequencies of foods consumed between those who did not know their serum phosphorus concentrations and those who did. Multiple factors may have impacted the participants’ ability to know their serum phosphorus concentrations including recency of last laboratory or clinic visit, the amount of laboratory information given to participants following a blood test, technological proficiency, or medical literacy [37]. Regardless of the reason a participant may not know their exact serum phosphorus concentration, increasing patient-focused education surrounding dialysis treatment can be utilized to address this issue and improve treatment outcomes. An observational study of healthy adults (*n* = 103) investigated their challenges in understanding laboratory results and provided educational information at different medical literacy levels. Results found that providing adults with both generic and personalized information about their laboratory results improved their comprehension [37]. Additionally, providing participants with educational resources, responding in a timely manner, and utilizing artificial intelligence to develop personalized recommendations can address adult challenges when it comes to interpreting laboratory results [37]. Specifically focusing on the impact of education among adults on dialysis, the RightStart program provided tailored information about various topics of interest and demonstrated a 41% decrease in the risk of death within the first 90 days of dialysis treatment [38]. These results highlight the importance of increasing both access to and dissemination of medical education and laboratory results to new or existing adults in the healthcare arena.

The results of this study demonstrated that certain aspects of participants’ PD or HD lifestyles may have impacted their dietary habits and phosphorus knowledge. Even though this was not asked on the questionnaire, if a participant had gastrointestinal issues, lack of appetite, or other physiological/physical issues that impacted their eating habits, they may have consumed foods that contained inorganic phosphates regardless of their phosphorus knowledge. A study investigated the causes of poor dietary choices in individuals with adequate nutrition knowledge and discussed how factors such as personal food taste preference, cultural food consumption, poor mental health, financial stress, lack of access to a healthy grocery store, and environmental temptations can lead to poor food choices even if knowledge is adequate [39]. Further studies should focus not only on knowledge of specific nutrients for adults undergoing dialysis, but also reasons why they consume certain foods.

### Limitations 

The cross-sectional survey was administered and accessed solely online, so people who did not have access to the internet could not be sampled. Additionally, the online survey was sent to adults undergoing dialysis who were active on online forums and threads, potentially neglecting adults on dialysis who may not partake of the use of these online resources. Individuals who engage in online activities regarding their dialysis may be more interested and knowledgeable about dialysis treatment in general, potentially leading to higher phosphorus knowledge scores than would be acquired from all adults on dialysis. Moreover, the phosphorus knowledge questionnaire was a multiple-choice test, allowing surveyed individuals to guess the answer to a question if they did not have the knowledge required. Additionally, the FFQ was phrased to assess phosphorus consumption over the period of a month, and individuals surveyed may not have accurately remembered what foods they consumed or how often they consumed the listed foods during that period. This ultimately would have led to a reporting bias that impacted the accuracy of their estimated daily average phosphorus intake. Finally, blood samples were not taken from the surveyed participants nor reviews of medical charts, and therefore the serum phosphorus levels that were provided by participants may not be accurate as their known serum level may not be the result of a recent laboratory test. 

## 5. Conclusions

The results of this cross-sectional study support the importance of managing serum phosphorus levels and increasing phosphorus education for adults undergoing dialysis in the US. The knowledge scores on the phosphorus knowledge questionnaire were adequate but on the lower end, indicating a gap in understanding and education that could have a major impact on treatment outcome and duration for those undergoing PD or HD. This study also encountered barriers that certain adults on dialysis must overcome, such as a lack of knowledge of their serum phosphorus levels or inadequate dietary choices that may increase their risk of cardiovascular or cerebrovascular events. While demographic factors and variations were not significantly correlated to phosphorus knowledge or consumption, differences between HD and PD treatment should be considered when developing a treatment protocol or regime aimed to control daily phosphorus intake and preserve renal function.

Increasing the sample size with both online surveys as well as in-person surveys in dialysis clinics could be beneficial for future studies to reach a more diverse population. Furthermore, alternative dietary assessment methods could be used to keep track of food intake such as diet records/food diaries, 24 h recalls, food frequency questionnaires, or a combination to reach more accurate consumption results. Lastly, future research can expand to other minerals of interest such as potassium, calcium, and sodium to study their impact on the health of adults on dialysis.

## Figures and Tables

**Table 1:** Demographics of participants (*n* = 107).

Demographic Variables	Total Participants (*n* = 107)	PD Participants (*n* = 61)	HD Participants (*n* = 46)
	N (%)	N (%)	N (%)
Dialysis vintage	106	60	47 *
1–2 months	7 (6.6%)	6 (10.0%)	2 (4.3%)
3–6 months	12 (11.3%)	7 (11.7%)	5 (10.6%)
7–12 months	17 (16.0%)	12 (20.0%)	5 (10.6%)
1–2 years	28 (26.4%)	20 (33.3%)	8 (17.0%)
2–4 years	15 (14.2%)	7 (11.7%)	8 (17.0%)
4 years or more	27 (25.5%)	8 (13.3%)	19 (40.5%)
Race/Ethnicity	104	57	46
African-American	8 (7.7%)	6 (10.5%)	2 (4.3%)
American Indian/Native/Alaskan Native	3 (2.9%)	1 (1.8%)	1 (2.2%)
Asian	9 (8.6%)	4 (7.0%)	5 (10.9%)
Hispanic	2 (1.9%)	2 (3.5%)	0 (0%)
White	81 (77.9%)	43 (75.4%)	38 (82.6%)
Prefer not to say	1 (1.0%)	1 (1.8%)	0 (0%)
Education Level	107	60	47
Some high school	0 (0%)	0 (0%)	1 (2.1%)
High school diploma or GED	13 (12.1%)	7 (11.7%)	6 (12.8%)
Some college	31 (28.9%)	17 (28.3%)	14 (29.8%)
Associates or tech degree	17 (15.9%)	9 (15.0%)	8 (17.0%)
Bachelor’s	27 (25.2%)	13 (21.7%)	13 (27.7%)
Graduate or Professional	17 (15.9%)	12 (20.0%)	5 (10.6%)
Prefer not to say	2 (2.0%)	2 (3.3%)	0 (0%)
Annual household income (USD)	106	60	46
Less than 25,000	23 (21.5%)	11 (18.3%)	12 (26.1%)
25,000–49,000	15 (14.0%)	9 (15.0%)	6 (13.0%)
50,000–74,999	16 (15.0%)	7 (11.7%)	8 (17.4%)
75,000–99,999	8 (7.5%)	5 (8.3%)	2 (4.3%)
100,000–149,999	18 (16.8%)	9 (15.0%)	9 (19.6%)
150,000 or more	11 (10.3%)	10 (16.7%)	1 (2.2%)
Prefer not to say	16 (15.0%)	8 (13.3%)	8 (17.4%)
Discussed phosphorus	107	60	47
Yes	102 (95.3%)	58 (96.7%)	44 (93.6%)
No	5 (4.7%)	2 (3.3%)	3 (6.4%)
Frequency of phosphorus discussion	107	60	47
Daily	1 (0.9%)	0 (0%)	1 (2.1%)
Once a week	8 (7.5%)	4 (6.7%)	4 (8.5%)
Once a month	60 (56.1%)	38 (63.3%)	22 (46.8%)
3–6 months	26 (24.3%)	12 (20.0%)	14 (29.8%)
12 months or longer	12 (11.2%)	6 (10.0%)	6 (12.8%)

Note. * indicates a significant difference in dialysis vintage between PD and HD participants based on chi-square test (*p* = 0.02).

**Table 2:** Independent *t*-test comparing PD group (*n* = 60) to the HD group (*n* = 47) for knowledge scores and dietary intake of phosphorus.

Variables	Peritoneal		Hemodialysis		*p* *
	M	Range	M	Range	
Dietary Intake of Phosphorus	625	542.5–706.9	576	505.8–646.1	0.00
Total Phosphorus Knowledge Score out of 19	10.4	9.7–11.1	11.0	10.2–11.9	0.00

Note. *p* * indicates statistical significance.

**Table 3:** Spearman Correlation with Phosphorus Knowledge Scores and Dietary intake of Phosphorus with Demographic Variables (*n* = 107).

	Discussed Phos. ^1^	Frequency of Discussing Phos.	Knowledge of Serum Phos.	Phosphate Binders	Race/ Ethnicity	Education Level	Income
Dietary Intake of Phosphorus	−0.07	−0.03	−0.18	0.02	0.10	−0.10	−0.02
Knowledge Score	0.21 *	−0.03	0.44 **	0.08	−0.02	0.13	0.01

^1^ Phos = phosphorus, * *p* < 0.05; ** *p* < 0.01.

## Data Availability

The original contributions presented in the study are included in the article, further inquiries can be directed to the corresponding author/s.
